# CHARACTERIZATION OF AGE-RELATED BIOENERGETIC DECLINE IN HUMAN DERMAL FIBROBLASTS

**DOI:** 10.1093/geroni/igad104.2482

**Published:** 2023-12-21

**Authors:** Howard Phang, Stephanie Heimler, Anthony Molina

**Affiliations:** University of California, San Diego, San Diego, California, United States; UC San Diego School of Medicine, San Diego, California, United States; University of California, San Diego, San Diego, California, United States

## Abstract

Progressive mitochondrial dysfunction is a widely recognized hallmark of the human aging process. However, efforts to model bioenergetic decline in vitro largely fail to recapitulate the context of human aging and metabolic physiology, making investigation of age-related bioenergetic decline and targeted interventions difficult. We sought to model human bioenergetic decline in vitro by profiling the respiratory capacity of cultured human dermal fibroblasts from donors across the adult human lifespan. Primary human dermal fibroblasts (pHDF) are an attractive in vitro model to assess age-related bioenergetic decline due to retention of key genotypic and phenotypic features of the donor during cell culturing, including age-related phenotypes. We hypothesized that respiratory capacity of pHDF is associated with the chronological age of the donor. We leveraged the San Diego Nathan Shock Center of Excellence (SD-NSC) study which is collecting pHDF from healthy adult (aged 20-85+) participants representative of “normal” aging based on strict inclusion and exclusion criteria. We profiled the mitochondrial function of eleven SD-NSC pHDF lines using the Seahorse XFe96 Mito Stress Test. The Pearson correlation identified a strong, negative correlation between maximal respiration and age (r= –0.69, p=0.02) and between spare respiratory capacity and age (r= –0.68, p=0.02). This suggests that human age-related mitochondrial dysfunction can be recapitulated in vitro using primary cell culture. These findings serve as the starting point for deeper analysis involving a larger sample of pHDF lines and other bioenergetic parameters to accurately quantify the effect of human aging on mitochondrial function and bioenergetics.

